# Lung adenocarcinoma with giant cyst formation showing a variety of histologic patterns: a case report

**DOI:** 10.1186/1752-1947-4-377

**Published:** 2010-11-25

**Authors:** Takeshi Kondo

**Affiliations:** 1 Division of Legal Medicine, Department of Community Medicine and Social Healthcare Science, Kobe University Graduate School of Medicine, 7-5-1 Kusunoki-cho, Chuo-ku, Kobe 650-0017, Japan

## Abstract

**Introduction:**

Lung cancer with large cyst formation is relatively rare. This is a case report of a patient with lung cystic adenocarcinoma with multiple histologic patterns. This type of lung adenocarcinoma is believed to be the first reported case in English language medical literature.

**Case presentation:**

A 60-year-old Japanese woman was admitted to hospital complaining of dyspnea and died of respiratory failure. She had been suffering from lung cancer with pleural effusion for five years. Autopsy analysis revealed lung adenocarcinoma with large cyst formation showing a variety of histologic patterns.

**Conclusions:**

Autopsy analysis of this atypical case of lung cancer may provide insight and lead to a better understanding of the heterogeneity and clonal expansion of lung adenocarcinoma.

## Introduction

Lung adenocarcinoma with large cyst formation is relatively rare and only a few cases have been reported [1-6]. This report is from an autopsy of a patient with lung adenocarcinoma with large cyst formation showing a variety of histologic patterns. It is believed that this type of lung cancer has not been reported in English language medical literature.

## Case presentation

A 60-year-old Japanese woman was admitted to hospital complaining of dyspnea. She had lung cancer with pleural effusion. A tumor was identified in the middle lobe of her right lung with pleural effusion. At that time, cytological examination was performed on the pleural effusion and the tumor was diagnosed as a conventional adenocarcinoma (the cytological specimen is not available). The pleural effusion had been controlled by drainage and chemotherapy. The lesion on her right lung showed an atypical appearance with giant pseudocyst formation, probably containing pleural effusion. She died of respiratory failure. An autopsy was immediately conducted to determine the pathological character of the pulmonary lesion with an atypical appearance.

**Figure 1:**
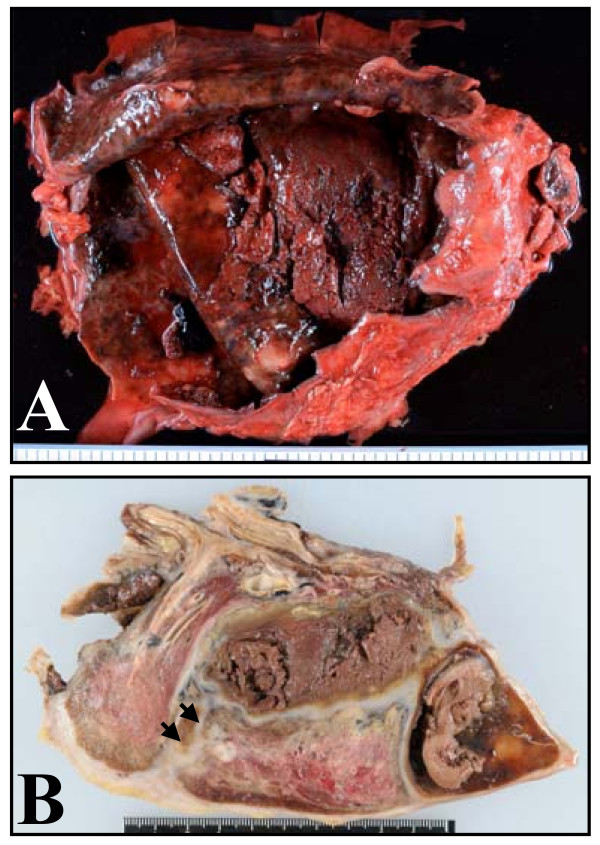
**Macroscopic findings of the tumor**. **A: **a 23 cm giant cyst occupying and adhered to the right thoracic cavity and containing necrotic material; **B: **pulmonary parenchyma compressed by the cyst (cut surface after fixation). Several tumor nodules were found (arrows).

**Figure 2:**
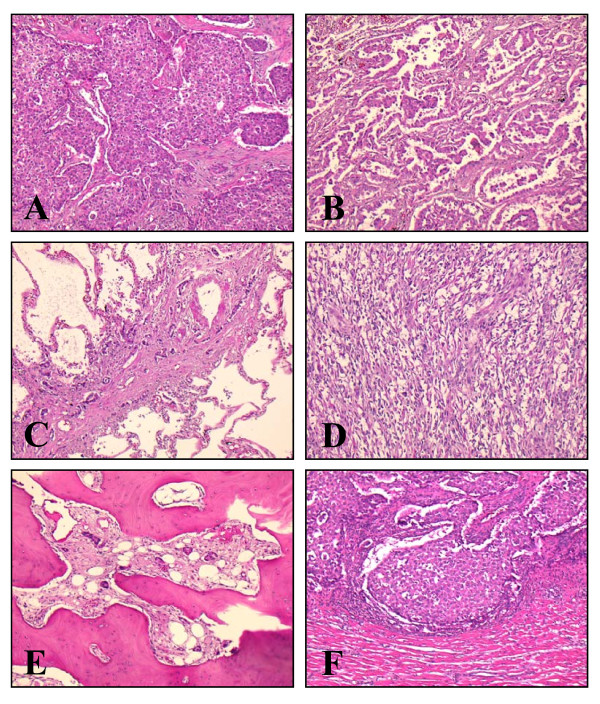
**Microscopic findings of the tumor (HE ×100)**. **A: **Solid pattern (major component of the tumor); **B: **bronchioloalveolar papillary pattern; **C: **severe lymphatic invasion; **D: **sarcomatous pattern with immunohistochemical expression of epithelial marker (not shown); **E: **paucicellular osteoblastic pattern; **F: **metastasis involving the myocardium.

Macroscopically, her right lung, weighing 655 g, had a large cyst containing pleural effusion (950 ml) and necrotic tissue (385 g) (Figure 1A, B). Aggressive metastasis was confirmed at various loci including her left lung, myocardium (Figure 2F), left adrenal gland, subcutaneous tissue around her right humerus, and bone (Figure 2E). Bone metastases were found in her lumbar vertebrae (L4/5) and the distal end of her right humerus which had a pathological fracture.

Microscopically, the tumor showed various histologic patterns including a solid pattern (the main component of the tumor, Figure 2A), a bronchioloalveolar papillary pattern (Figure 2B), lymphatic invasion (Figure 2C), a sarcomatous pattern (Figure 2D), and a paucicellular osteoblastic pattern n the vertebral metastatic site (Figure 2E). A sarcomatous component was found in the mural nodule of the cyst and it was revealed on immunohistochemistry that it was positive for cytokeratin (clone: CAM5.2, image not shown).

## Discussion

This intriguing case showed various histologic patterns, including a solid pattern (the main component), a bronchioloalveolar papillary pattern, lymphatic invasion, a sarcomatous pattern, and a hypocellular osteoblastic pattern.

Only a few cases of lung carcinoma with cyst formation have been reported [1-6]. The possible mechanisms for the development of malignant cysts include necrosis of the central core of the tumor followed by discharge of the necrotic content [6]. In this case report, the large cyst contained only necrotic material and no viable cells were included, although the viability of the tumor nodule itself was very high.

Etiothanatopathological (here, *etiothanatopathology *has been coined from *etio*logy, *thanato*logy, and *patho*logy) analysis of autopsy cases is important. The autopsy findings may give an insight into and lead to a better understanding of the heterogeneity and clonal expansion of lung adenocarcinoma.

## Conclusions

This is a case report of a patient with lung cystic adenocarcinoma with multiple histologic patterns. This rare case is believed to be the first reported case in English language medical literature. Analysis of lung cancer by autopsy may contribute to elucidation of the heterogeneity and clonality of lung adenocarcinoma.

## Consent

Written informed consent was obtained from the patient's next of kin for publication of this case report and any accompanying images. A copy of the written consent is available for review by the Editor-in-Chief of this journal.

## Competing interests

The author declares that they have no competing interests.
